# Study of Pharmacodynamic and Pharmacokinetic Interaction of Bojungikki-Tang with Aspirin in Healthy Subjects and Ischemic Stroke Patients

**DOI:** 10.1155/2018/9727240

**Published:** 2018-01-23

**Authors:** Jung-Hwa Yoo, Sung-Vin Yim, Byung-Cheol Lee

**Affiliations:** ^1^Department of Internal Medicine, College of Korean Medicine, Kyung Hee University, 23 Kyungheedae-ro, Dongdaemun, Seoul 02447, Republic of Korea; ^2^Department of Pharmacology, School of Medicine, Kyung Hee University, 23 Kyungheedae-ro, Dongdaemun, Seoul 02447, Republic of Korea

## Abstract

**Background:**

Bojungikki-tang (BJIKT) is a widely used traditional herbal formula in China, Japan, and Korea. There have been reports that several herbs among BJIKT have interactions with antiplatelet drugs, such as aspirin. This study aimed to assess whether BJIKT interacts with aspirin in terms of pharmacokinetics (PK) and pharmacodynamics (PD) in healthy subjects and ischemic stroke patients.

**Methods:**

The phase I interaction trial was a randomized, open-label, crossover study of 10 healthy male subjects, and the phase III interaction trial was a randomized, placebo-controlled, parallel study of 43 ischemic stroke patients. Each participant randomly received aspirin + BJIKT or aspirin + placebo. For PK analysis, plasma acetyl salicylic acid (ASA) and salicylic acid (SA) were evaluated, and, for PD analysis, platelet aggregation and plasma thromboxane B_2_ (TxB_2_) were measured.

**Results:**

In the PK parameters, mean area under curve, maximum concertation, and peak concentration time of ASA and SA were not different between two groups in healthy subjects and ischemic stroke patients. In the PD profiles, TxB_2_ concentrations and platelet aggregation were not affected by coadministration of BJIKT in healthy subjects and ischemic stroke patients.

**Conclusions:**

These results suggest that coadministration of BJIKT with aspirin may not result in herb-drug interaction.

## 1. Introduction

The use of combination of herbal supplements and modern drugs has become increasingly popular in recent years [1]. Although this combined therapy has been reported to be beneficial in strengthening therapeutic effects or reducing side-effects, numerous reports exist in which negative, undesired effects have been reported [1, 2].

A drug–herb interaction can be defined as pharmacologic or clinical response to the coadministration of modern pharmaceutical drugs and herbal products [3]. The high prevalence of both conventional pharmacological therapy and herbal medicines use draws attention to safety concerns. However, the prevalence of drug–herb interactions is unknown, signifying the negligence of the consumers in reporting adverse herb reaction or drug–herb interactions. [3, 4].

Bojungikki-tang (BJIKT; Bu-Zhong-Yi-Qi-Tang in Chinese or Hochu-ekki-to in Japanese) is a widely used traditional herbal formula in China, Japan, and Korea. BJIKT, originally meaning “Tonify the Middle and Augment the Qi Decoction,” has been identified as an effective medication to improve conditions such as general fatigue and poor appetite and as an adjunct to treating stroke caused by qi deficiency and treating debilitating condition resulting from sequelae of cerebrovascular disease [5–8]. There have been reports that several medicinal herbs among BJIKT composed natural herbs have interactions with antiplatelet drugs, such as aspirin [9–14].

However, to date, there have been no randomized clinical trials focusing on herb-drug interaction. Accordingly, the objective of this study was to assess whether BJIKT interacts with an antiplatelet drug in terms of pharmacokinetics (PK) and pharmacodynamics (PD) in healthy individuals as well as in ischemic stroke patients.

## 2. Materials and Methods

### 2.1. Subjects

The phase I study population consisted of 10 healthy adult male volunteers. The mean age was 25.4 ± 3.4 years (20–33 years). The mean body weight was 70.9 ± 10.2 kg and the mean height was 175.5 ± 4.9 cm. Significant exclusion criteria for study included a history of allergy to aspirin or herbal medication; history of renal, hepatic, cardiovascular, gastrointestinal, or neurologic diseases that might significantly alter the absorption, distribution, metabolism, and excretion of the study drug; known hypersensitivity to the study drugs; acute disease within the past 28 days from the administration of a drug; participation in another clinical study within the past 60 days; receiving medications that induce or inhibit drug-metabolizing enzymes, such as barbiturates, within past 30 days; history of excessive drinking; illiteracy; or inability to be protected by parental rights.

The phase III study population was selected from 322 ischemic stroke patients over 40 years old (range 41–77 years) who were living in Korea. Prospective participants were screened at Kyung Hee University Medical Center from March 2010 to April 2011. Of these, 43 subjects participated in this study. The inclusion criteria included individuals taking aspirin for over 3 months with a previous diagnosis of ischemic stroke, defined as an acute focal or global neurological deficit lasting more than 24 hours without an apparent cause other than vascular origin, consecutively confirmed by magnetic resonance imaging (MRI) within 72 hours of the onset of symptoms. Patients with cerebral hemorrhage, cerebral venous thrombosis, or a brain tumor and those who met any of the phase I exclusion criteria were excluded. Of the 43 ischemic stroke patients enrolled in phase III, 39 subjects completed the study and were included in PK and immunogenicity analyses (17, aspirin + BJIKT; 22, aspirin + placebo). Four subjects from the aspirin + placebo group and 0 subjects in the aspirin + BJIKT group withdrew consent after receiving the study drug. The baseline demographic characteristics of subjects in each group were well balanced within the groups (Table 1).

Both phase I and phase III studies were approved by the institutional review board of Kyung Hee University Medical Center (phase I: KMC IRB 0917-03-A2; phase III: KOMC IRB 2009-15) and were also approved by Korean Food Drug Administration (KFDA) (phase I: 2010-135; phase III: 2009-1080). Written informed consent was obtained from each participant, and studies were conducted in accordance with the principles of the International Conference of Harmonization for Good Clinical Practice (ICH-GCP) and the ethical standards for human experimentation established in the Declaration of Helsinki. The study was registered with Clinical Research Information Service (CRIS): KCT0002049.

### 2.2. Study Drugs

We purchased the BJIKT extract granules that contain a mixture of spray-dried hot water extracts of 10 medicinal plants from Hanpoong Pharmacy & Foods Company (Seoul, Korea). The 10 medical plants are Astragali radix (16.7%), Atractylodis lanceae rhizoma (16.7%), Ginseng radix (16.7%), Angelicae radix (12.5%), Bupleuri radix (8.3%), Zizyphi fructus (8.3%), Aurantii nobilis pericarpium (8.3%), Glycyrrhizae radix (6.3%), Cimicifugae rhizoma (4.2%), and Zingiberis rhizoma (2.0%). A voucher specimen (code number HX018) was deposited in herbarium in the department of herbal pharmacy, Kyung Hee Korean Medical Hospital.

Each herb in BJIKT was quality controlled from the places of origin to the final products. The active ingredients were also quality controlled by using high-performance liquid chromatography. According to the compilation of specification and test procedures of Hanpoong Pharmacy & Foods Company, BJIKT contains 52.0 mg of hesperidin (C_28_H_34_O_15_ in Citri Unshii Pericarpium), 5.4 mg of ginsenoside Rb_1_ (C_54_H_92_O_23_ in Ginseng radix), 50.0 mg of decursin (C_19_H_20_O_5_ in Angelicae Gigantis Radix), 6 mg of zingerol (C_17_H_26_O_4_ in Zingiberis Rhizoma), and 67.5 mg of glycyrrhizic acid (C_42_H_62_O_16_ in Glycyrrhizae Radix et Rhizoma) per pack.

And enteric-coated tablet aspirin (Aspirin Cardio™) 100 mg was donated from Bayer HealthCare Pharmaceuticals, Germany.

### 2.3. Protocol

The phase I interaction study was conducted in the Kyung Hee Clinical Research Institute, Kyung Hee Medical Center, Kyung Hee University (Seoul, Korea). It was a randomized, open-label, crossover study of 10 healthy male subjects. A day before the study, eligible subjects were hospitalized in the Kyung Hee Clinical Research Institute. After overnight fasting, each subject randomly received an oral administration of 3 packs of BJIKT or placebo (1 pack volume: 6.83 g) at 7 AM and additionally given the 2 capsules of Aspirin Cardio 100 mg (Bayer HealthCare Pharmaceuticals, Germany) at 8 AM with 240 ml tab water. For the pharmacokinetic analysis, blood acetyl salicylic acid and salicylic acid were measured 0, 5, 10, 20, 30, 45, 60, 90, 120, 180, 240, 360, 480, and 600 min after aspirin administration. For pharmacodynamic analysis, platelet aggregation was measured 0, 2, and 4 h, and plasma thromboxane B2 (TxB2) was measured 0, 5, 10, 20, 30, 45, and 60 min after aspirin administration. After 1 week wash-out period, administration of each group was exchanged, and all measurements were repeated in the same manner.

The phase III interaction study was conducted at Kyung Hee Korean Medical Hospital, Kyung Hee University (Seoul, Korea). It was a randomized, placebo-controlled, double-blinded, parallel study. Eligible ischemic stroke patients were randomly allocated to either BJIKT treatment or placebo, in addition to aspirin. After allocation, each subject randomly received oral administration of BJIKT or placebo 3 times a day (1 pack volume: 6.83 g) as well as 1 capsule of Aspirin Cardio 100 mg every morning for 2 weeks [15]. For PK analysis, blood ASA and SA were measured 2 hours after aspirin administration at 0, 1, and 2 weeks. For PD analysis, platelet aggregation and plasma TxB_2_ were measured 1 hour after aspirin administration at 0, 1, and 2 weeks. At every visit, all of the laboratory tests done at screening as well as basic exams (weight, height) were repeated. Blood samples were collected for participant safety and outcome assessment. Participants were asked about adverse events, and BJIKT packs and aspirin capsules were counted to assess compliance (Table 5).

### 2.4. Measurement of Acetyl Salicylic Acid and Salicylic Acid

Plasma concentration of acetyl Salicylic acid (ASA) and salicylic acid (SA) which is the further metabolite of ASA was determined with liquid chromatography (UPLC, Waters Corp)/tandem mass spectrometry (Qtrap 5500, AB sciex). The compounds were separated using reverse column (ACQUITY UPLC C18 Column, 1.7 *μ*m, 2.1 × 50 mm) with an isocratic mobile phase consisting of acetonitrile and water (70 : 30, v/v; with 0.1% formic acid) at flow rate 0.2 ml/min. Detection was performed using electrospray ionization (ESI) source in the negative ion mode at −4500 eV and 600°C. The operating conditions were optimized for each of the analytes and were determined as follows: nebulizing gas (Gas1), 60; heater gas (Gas2), 60; curtain gas, 30. Quantification was performed by multiple-reaction monitoring (MRM). The masses for ASA and SA were* m/z* 179 → 135 (with declustering potential −80, collision energy −16) and 136.8 → 93 (with declustering potential −140, collision energy −15.5). The internal standard of this study was simvastatin and the mass was* m/z* 434.6 → 367 (with declustering potential −60, collision energy −15). The lower limit of quantification (LLOQ) for ASA and SA was 5 ng/mL and 50 ng/mL, respectively.

Pharmacokinetic parameters for ASA and SA were determined including *C*_max_ (maximum plasma concentration), *T*_max_ (time point of maximum plasma concentration), and AUC_0–∞_ (area under the plasma concentration versus time curve from 0 h to infinity).

### 2.5. Measurement of Platelet Aggregation and Plasma Thromboxane B2 Level

Platelet aggregation experiments were performed using Chrono-Log model 700 two-channel whole Blood/Optical Lumi-Aggregometer (Chrono-Log Corporation). Platelet-rich plasma (PRP) was obtained from the citrated blood centrifugation (Beckman Allegra 6R) at 800 rpm for 10 min. Platelet-poor plasma (PPP) was obtained from PRP centrifugation (eppendorf 5415R) at 13000 rpm for 10 min. Collagen (Chrono-Log Corporation) was used to induce the platelet aggregation. The change in absorbance was recorded until the response reached a plateau or for 5 min.

Plasma thromboxane B_2_ (TXB_2_) was measured using an enzyme immunoassay kit (Thromboxane B_2_ EIA Kit, Cayman Chemical, MI, USA). The standard curve was prepared as outlined in the manufacturer's instructions. The thawed test and control plasma samples were tested in duplicate.

### 2.6. Statistical Analysis

The study sample sizes were determined from variance estimates based on prior aspirin platelet aggregation PD data [15]. To evaluate clinically relevant interactions, we used the noninferiority approach with 90% test power and a two-sided alpha value of 0.05. Data were presented as the mean ± standard deviation. Baseline demographics and clinical variables in the phase III study were compared among treatment groups using a Student's *t*-test, chi-square test, or Fisher's exact test. The changes in PK or PD 1 and 2 weeks from baseline were compared between the BJIKT treatment group and the placebo group using a Student's *t*-test. All *P* values were two-tailed, and significance was set at *P* < 0.05. All statistical analyses were performed using the GraphPad Prism for Windows, Version 5.01 (GraphPad Software, Inc.).

## 3. Results

### 3.1. Pharmacokinetic Effects of Aspirin and Bojungikki-Tang Coadministration

**Figure 1:**
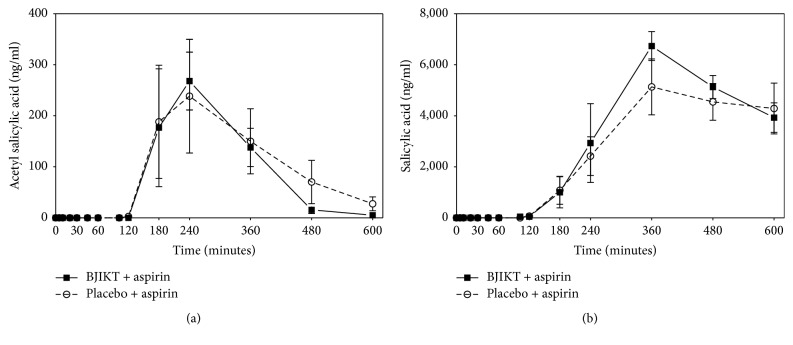
The pharmacokinetic profiles of healthy subjects (phase I study). Data are shown as mean and standard deviation of acetyl salicylic acid (a) and salicylic acid levels (b) in Bojungikki-tang (BJIKT) + aspirin and placebo + aspirin.

In the phase I trial with healthy subject, the area under curve (AUC) of acetyl salicylic acid in placebo group was 60959.5 ± 14243.3 ng/ml, while in BJIKT group it was 53465.3 ± 6777.6 ng/ml. Mean ± SE peak plasma concentration (*C*max) value in the placebo was 440.8 ± 100.8 ng/ml, while in the BJIKT it was 418.2 ± 81.5 ng/ml. Time to peak concentration (*T*max) of salicylic acid in placebo was 312.0 ± 43.6 min, while in the BJIKT it was 312.0 ± 24.9 min. There were no significant differences between two groups in mean values of AUC,* C*max, and* T*max (Table 2, Figure 1). The plasma concentrations of salicylic acid at various time intervals have been plotted in Figure 1. When an Aspirin was administrated with BJIKT, the area under the curve (AUC) of salicylic acid in placebo group was 1704207.8 ± 276367.4 ng/ml, while in BJIKT group it was 1986552.4 ± 252995.5 ng/ml. Mean ± SE peak plasma concentration (*C*max) value in the placebo was 6284.3 ± 1029.1 ng/ml, while in the BJIKT it was 7550.8 ± 906.2 ng/ml. Time to peak concentration (*T*max) of salicylic acid in placebo was 414.0 ± 44.2 min, while in the BJIKT it was 348.0 ± 21.5 min. There were no significant differences between two groups in mean values of AUC,* C*max, and* T*max (Table 2, Figure 1).

In the phase III trial with ischemic stroke patients, the plasma concentration of ASA with 100 mg of aspirin in the placebo group was decreased to be −15.3 ± 14.5 ng/ml at 2 weeks, compared with 27.8 ± 17.4 ng/ml at baseline. The ASA in the BJIKT group was decreased to be −18.3 ± 14.1 ng/ml at 2 weeks, compared with 19.1 ± 11.5 ng/ml at baseline (Table 4). However, there was no significant difference between the combination of aspirin with BJIKT and the placebo. When aspirin was administrated with BJIKT, the mean plasma SA concentration changed −374.1 ± 506.3 compared with baseline (2298.6 ± 598.2). In the placebo group, the mean plasma SA concentration decreased −244.1 ± 522.9 ng/ml at 2 weeks compared with baseline (2953.7 ± 571.2 ng/ml) (Table 4). A statistically significant change in SA was not detected in either the BJIKT or the placebo group.

### 3.2. Pharmacodynamic Effects of Aspirin and Bojungikki-Tang Coadministration

**Figure 2:**
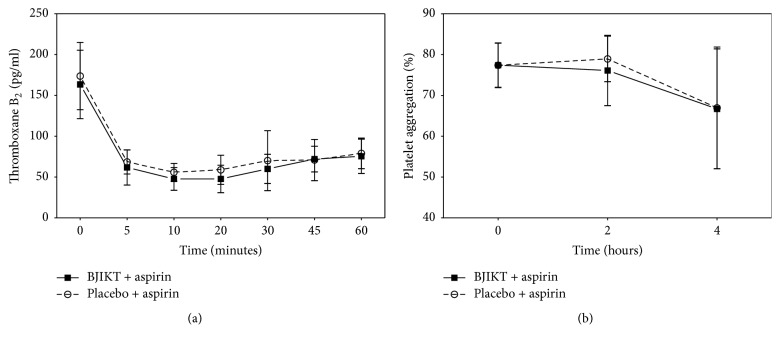
The pharmacodynamic profiles of healthy subjects (phase I study). Data are shown as mean and standard deviation of thromboxane B2 (a) and platelet aggregation (b) in Bojungikki-tang (BJIKT) + aspirin and placebo + aspirin.

In the phase I trial with healthy subject, when aspirin was administrated with a placebo, a 60.5% rapid decrease in mean plasma TxB_2_ concentrations compared with baseline was detected at 5 min (*P* < 0.001). In the BJIKT group, a 62.2% decrease in mean plasma TxB_2_ was also detected at 5 min (*P* < 0.001) (Table 3, Figure 2). A statistically significant decrease in plasma TxB_2_ was not detected in either the BJIKT or placebo groups at baseline or various time intervals. The inhibition of platelet aggregation at various time intervals for the BJIKT or placebo groups combined with aspirin was analyzed. In the placebo group, platelet aggregation with 300 mg of aspirin was found to be 66.7 ± 14.7% at 4 h compared with 77.4 ± 5.4% at baseline (*P* < 0.001). In the BJIKT group, the platelet was decreased to be 66.9 ± 14.9% at 4 h compared with 78.1 ± 5.4% at baseline (*P* < 0.001). However, the combination of aspirin with either BJIKT or a placebo did not potentiate the inhibition of collagen-induced platelet aggregation (Table 3, Figure 2).

In the phase III trial with ischemic stroke patients, the effects of the combination of aspirin with either BJIKT or placebo on plasma TxB_2_ concentrations are shown in Table 4. When aspirin was administrated with a placebo, the mean plasma TxB_2_ concentrations decreased −13.9 ± 2.5 pg/ml in 2 weeks compared with baseline (16.2 ± 3.4 pg/ml). In the BJIKT group, mean plasma TxB_2_ concentration changed −13.6 ± 3.6 pg/ml in 2 weeks (Table 4). However, a statistically significant decrease in plasma TxB_2_ was not detected in either the BJIKT or placebo groups. The inhibition of platelet aggregation at baseline and 1 and 2 weeks for the BJIKT and placebo groups combined with aspirin was accessed. In the placebo group, the platelet aggregation with 100 mg of aspirin was decreased to be −0.2 ± 5.1% at 2 weeks compared with 74.8 ± 3.6% at baseline. In the BJIKT group, the platelet aggregation was found to be −1.1 ± 2.8% at 2 weeks compared with 73.6 ± 2.5% at baseline. However, the combination of aspirin with either BJIKT or the placebo did not change the inhibition of collagen-induced platelet aggregation (Table 4).

## 4. Discussion

This is the first study to investigate the PK and PD profiles of the herb-drug interaction between BJIKT and aspirin among healthy subjects and patients with ischemic stroke. Overall, there were no apparent differences in the PK profiles of ASA and SA and PD profiles of TxB_2_ and platelet aggregation between aspirin alone and coadministration with BJIKT among either healthy subjects or ischemic stroke patients, suggesting that BJIKT may not interact with aspirin.

The increased use of herbal medicines and supplements worldwide has substantially increased the number of potential drug interactions with modern drugs, and there has been the documented experimental and clinical evidence of interactions between herbal products and modern drugs [9]. Among these, herbs that may influence the effect of antiplatelet treatment are of particular interest, considering that treatment for coronary artery diseases and ischemic stroke is quite common, and aspirin is one of the drugs most frequently used to treat these diseases.

BJT may be prescribed for stroke caused by qi deficiency. It is also a typical prescription for deficiency of middle qi and overall symptoms of qi deficiency [16]. This prescription may tonify the spleen and raise and circulate pure qi, which is effective against fever, heart discomfort, sweating, fatigue, dizziness, numbness, and weakness [16]. BJIKT is composed of 10 natural herbs, among which Ginseng inhibited TxA_2_ formation and thus platelet aggregation in* in vitro* studies [9, 17, 18] and impaired platelet aggregation in rats [19]. Astragalus could reduce platelet adhesion and aggregation, reduce plasma fibrinogen, and show antithrombus formation effect [10–12]. Glycyrrhizae can inhibit thrombin and platelet aggregation, therefore enhancing the risk of bleeding with antiplatelets and anticoagulants [4]. Hesperidin, which is major component of Citri Unshii Pericarpium, has been documented to inhibit TxB_2_ formation and human platelet aggregation [14]. However, there are controversial reports that Ginseng inhibited CYP2D6, but the magnitude of the effect did not appear clinically relevant [20], and Citri Unshii demonstrated a relatively low frequency of drug interactions and had weak inhibitory effects on CYP2C9, which metabolizes NSAIDs [21, 22]. Furthermore, cocktail herbal medicine including Ginseng and Citrus, not single herb, indicated no significant effect on CYP1A2, CYP2D6, CYP2E1, and CYP3A4 activity in healthy volunteers [20]. Therefore, our results, along with previous reports, support the idea that BJIKT does not affect the antiplatelet effects of aspirin.

In the PK study, *C*_max_ , *T*_max_, and AUC of plasma ASA and SA were not changed by BJIKT among healthy subjects and ischemic stroke patients. And PD profiles showed no apparent differences in the TxB_2_ and platelet aggregation between aspirin alone and coadministration with BJIKT among healthy subjects and even among ischemic stroke patients.

Most herb-drug interaction studies have used* in vitro* testing of herbal constituents in microsomal system or conducted in healthy subjects, but most relevant results have been obtained when conducted in the patients who have used the herb and drug together. Thus, the advantage of this study is conducted with patients as well healthy subjects. However, BJIKT consist of 10 medicinal plants and contain multiple compounds, which may not accurately represent all the effects of each plant and/or compound. Moreover, the sample size of this study is relatedly small and all participants are Korean, which may also have the limitation of applying study result to other races. Therefore, further study with large sample and various races should be required.

## 5. Conclusions

In conclusion, the PK and PD profiles of aspirin were not affected by combined treatment with BJIKT. No safety clinical concerns were raised among ischemic stroke patients. These results suggest that coadministration with BJIKT for the purpose of antiplatelet effects may not result in herb-drug interaction.

## Figures and Tables

**Table 1:** Baseline characteristics of healthy subjects and ischemic stroke patients.

	Healthy subjects (*n* = 10)	Ischemic stroke		
		Aspirin + placebo (*n* = 21)	Aspirin + BJIKT (*n* = 22)	*P*
Age	28.7 ± 4.2	60.0 ± 10.9	64.72 ± 7.1	NS
Male (*n*, %)	10 (100)	13 (61.9)	14 (63.6)	NS
BMI (kg/m^2^)	23.7 ± 1.7	24.7 ± 3.1	24.1 ± 1.7	NS
Heart disease (*n*, %)	0	3 (14.2)	7 (31.8)	NS
HBP (*n*, %)	0	13 (61.9)	14 (63.6)	NS
Diabetes (*n*, %)	0	6 (28.5)	6 (27.2)	NS
Dyslipidemia (*n*, %)	0	10 (47.6)	9 (40.9)	NS
Smoking (*n*, %)	0	2 (9.5)	2 (9.1)	NS
Alcohol (*n*, %)	0	7 (33.3)	8 (36.3)	NS

BJIKT: Bojungikki-tang, BMI: body mass index, and HBP: high blood pressure.

**Table 2:** Pharmacokinetic parameters among healthy subjects (phase I study).

	ASA			SA		
	Aspirin + placebo	Aspirin + BJIKT	*P*	Aspirin + placebo	Aspirin + BJIKT	*P*
AUC_0–last_ (ng m/ml)	60959.5 ± 14243.3	53465.3 ± 6777.6	N.S.	1704207.8 ± 276367.4	1986552.4 ± 252995.5	NS
*C*_max_ (ng/ml)	440.8 ± 100.8	418.2 ± 81.5	N.S.	6284.3 ± 1029.1	7550.8 ± 906.2	NS
*T*_max_ (min)	312.0 ± 43.6	312.0 ± 24.9	N.S.	414.0 ± 44.2	348.0 ± 21.5	NS
*t*_1/2_ (min)	68.4 ± 21.6	50.1 ± 10.7	N.S.	319.2 ± 118.6	252.0 ± 57.4	NS

BJIKT: Bojungikki-tang, ASA: acetyl salicylic acid, SA: salicylic acid, AUC_0–last_: area under the serum concentration-time curve from time 0 to 10 hours after aspirin administration, *C*_max_: maximum plasma concentration, *T*_max_: time point of maximum plasma concentration, and *t*_1/2_: terminal elimination half-life.

**Table 3:** Pharmacodynamic parameters among healthy subjects (phase I study).

	Aspirin + placebo	Aspirin + BJIKT	*P*
TxB_2_ (pg/ml)			
0 min	173.6 ± 41.2	163.4 ± 41.8	NS
5 min	68.5 ± 14.8	61.7 ± 21.5	NS
10 min	55.81 ± 10.9	47.6 ± 13.9	NS
20 min	58.8 ± 17.8	47.6 ± 16.8	NS
30 min	70.1 ± 36.7	59.9 ± 17.8	NS
45 min	70.9 ± 25.2	71.9 ± 15.7	NS
60 min	78.9 ± 18.7	75.4 ± 21.1	NS
PLT agg (%)			
0	77.4 ± 5.4	78.1 ± 5.4	NS
2 hours	76.1 ± 8.6	78.9 ± 5.5	NS
4 hours	66.7 ± 14.7	66.9 ± 14.9	NS

BJIKT: Bojungikki-tang, TxB_2_: thromboxane B_2_, PLT agg: platelet aggregation.

**Table 4:** Change in pharmacokinetic and pharmacodynamic profiles and blood tests among ischemic stroke patients (phase III study).

	Aspirin + placebo		Aspirin + BJIKT		*P*
	Baseline	Net change from baseline	Baseline	Net change from baseline	
PK					
ASA	27.8 ± 17.4	−15.3 ± 14.5	19.1 ± 11.5	−18.3 ± 14.1	NS
SA	2953.7 ± 571.2	−244.1 ± 522.9	2298.6 ± 598.2	−374.1 ± 506.3	NS
PD					
TxB_2_ (pg/ml)	16.2 ± 3.4	−13.9 ± 2.5	21.1 ± 5.3	−13.6 ± 3.6	NS
PLT agg (%)	74.8 ± 3.6	−0.2 ± 5.1	73.6 ± 2.5	−1.1 ± 2.8	NS
Blood chemistry					
FBG (mg/dl)	117.9 ± 39.8	−0.4 ± 52.1	120.8 ± 48.7	−9.2 ± 59.9	NS
T-chol (mg/dl)	171.0 ± 41.7	30.8 ± 141.4	171.1 ± 38.5	−5.1 ± 22.9	NS
TG (mg/dl)	153.7 ± 82.3	−17.2 ± 68.5	161.7 ± 69.9	−5.3 ± 57.4	NS
Ca	9.2 ± 0.3	5.1 ± 21.2	9.1 ± 0.2	4.0 ± 18.1	NS
P	3.1 ± 0.6	0.01 ± 0.4	3.1 ± 0.5	0.08 ± 0.4	NS
Uric acid	5.5 ± 1.5	0.1 ± 0.6	5.4 ± 1.6	−0.3 ± 0.6	NS
Blood count					
WBC	6.5 ± 1.0	−0.1 ± 1.4	6.8 ± 1.1	−0.1 ± 0.5	NS
RBC	4.7 ± 0.5	0.4 ± 1.4	4.5 ± 0.5	0.0 ± 0.2	NS
Hgb	14.1 ± 1.6	0.0 ± 0.6	13.8 ± 1.5	0.1 ± 0.5	NS
Hct	41.4 ± 4.4	0.1 ± 1.7	40.6 ± 4.6	0.2 ± 1.9	NS
Platelet	277.1 ± 82.2	10.8 ± 24.1	263.7 ± 69.9	9.9 ± 37.6	NS

BJIKT: Bojungikki-tang, ASA: acetyl salicylic acid, SA: salicylic acid, TxB_2_: thromboxane B_2_, PLT agg: platelet aggregation, FBS: fasting blood glucose, T-chol: total cholesterol, TG: triglyceride, Ca: calcium, P: phosphorus, WBC: white blood cell, RBC: red blood cell, Hgb: hemoglobin, and Hct: hematocrit.

**Table 5:** Safety parameters among ischemic stroke patients (phase III study).

	Aspirin + placebo		Aspirin + BJIKT		*P*
	Baseline	Net change from baseline	Baseline	Net change from baseline	
Liver function					
AST	24.7 ± 4.9	1.8 ± 5.9	25.2 ± 5.4	2.0 ± 6.3	NS
ALT	23.5 ± 11.7	1.2 ± 8.6	21.4 ± 8.1	1.6 ± 6.2	NS
GGT	29.6 ± 12.5	0.0 ± 4.4	29.1 ± 16.2	0.1 ± 4.9	NS
T-bil	0.6 ± 0.2	−0.0 ± 0.1	0.6 ± 0.2	0.0 ± 0.1	NS
ALP	78.1 ± 16.9	0.7 ± 7.4	68.5 ± 15.8	0.8 ± 6.2	NS
Protein	7.6 ± 0.5	0.0 ± 0.4	7.4 ± 0.4	−0.0 ± 0.4	NS
Albumin	4.4 ± 0.1	−0.0 ± 0.2	4.3 ± 0.2	−0.0 ± 0.1	NS
Kidney function					
BUN	15.6 ± 2.5	−1.8 ± 4.1	16.6 ± 5.3	−2.3 ± 4.7	NS
Cr	0.7 ± 0.1	−0.0 ± 0.0	0.8 ± 0.4	−0.0 ± 0.0	NS
Adverse effects					
Indigestion	1		1		NS
Diarrhea	1		0		NS

BJIKT: Bojungikki-tang, AST: aspartate aminotransferase, ALT: alanine aminotransferase, GGT: gamma-glutamyl transferase, T-bil: total bilirubin, ALP: alkaline phosphatase, and BUN: blood urea nitrogen.

## References

[1] Asher G. N., Corbett A. H., Hawke R. L. (2017). Common herbal dietary supplement-drug interactions. *American Family Physician*.

[2] de Lima Toccafondo Vieira M., Huang S.-M. (2012). Botanical-drug interactions: a scientific perspective. *Planta Medica*.

[3] Fugh-Berman A. (2000). Herb-drug interactions. *The Lancet*.

[4] Tachjian A., Maria V., Jahangir A. (2010). Use of herbal products and potential interactions in patients with cardiovascular diseases. *Journal of the American College of Cardiology*.

[5] Wang X. Q., Takahashi T., Zhu S. (2004). Effect of Hochu-ekki-to (TJ-41), a japanese herbal medicine, on daily activity in a murine model of chronic fatigue syndrome. *Evidence-Based Complementary and Alternative Medicine*.

[6] In-Seon Choi J.-N. K., Young-Kyun K. (2009). Neurological effects of bojungikki-tang and bojungikki-tang-gamibang on focal cerebral ischemia of the MCAO rats. *The Journal of Korean Oriental Medicine*.

[7] Kuratsune H. (1997). Effect of Kampo Medicine, "Hochu-ekki-to", on chronic fatigue syndrome. *Clinic and Research*.

[8] Scheid V., Bensky D., Ellis A., Barolet R. (2009). *Chinese herbal medicine: formulas strategies*.

[9] Abebe W. (2002). Herbal medication: potential for adverse interactions with analgesic drugs. *Journal of Clinical Pharmacy and Therapeutics*.

[10] Gao J., Xu X., Ni S. (2002). Research on antithrombotic effect of total saponins of astragalus. *Chinese Traditional Patent Medicine*.

[11] Xu Y., Gao P., Liang Q. (1999). Experimental study on effect of astragalus polysaccharide to the cerebral thrombosis. *Chinese Journal of Hematology*.

[12] Wang Q., Li J., Liu Y. (2003). Effect of astragalus injection on the formation of venous thrombosis in rats. *Chinese Traditional Patent Medicine*.

[13] Wu Y., Ouyang J., Tu S. (2002). Effects of Astragalus Polysaccharides on atherosclerosis endothelial cell injury. *Journal of Hubei College of Traditional Chinese Medicine*.

[14] Kim T.-H., Kim H.-M., Park S. W., Jung Y.-S. Inhibitory effects of yuzu and its components on human platelet aggregation. *Biomolecules & Therapeutics*.

[15] Becker D. M., Segal J., Vaidya D. (2006). Sex differences in platelet reactivity and response to low-dose aspirin therapy. *The Journal of the American Medical Association*.

[16] Heo J. (1999). *Translated Dongeuibogam*.

[17] Gruenwald J., Brendler T., Jaenicke C. (1998). *PDR for Herbal Medicines*.

[18] Kuo S.-C., Teng C.-M., Lee J.-C., Ko F.-N., Chen S.-C., Wu T.-S. (1990). Antiplatelet components in panax ginseng. *Planta Medica*.

[19] Park H.-J., Lee J.-H., Song Y.-B., Park K.-H. (1996). Effects of dietary supplementation of lipophilic fraction from Panax ginseng on cGMP and cAMP in rat platelets and on blood coagulation. *Biological & Pharmaceutical Bulletin*.

[20] Gurley B. J., Gardner S. F., Hubbard M. A. (2005). Clinical assessment of effects of botanical supplementation on cytochrome P450 phenotypes in the elderly: St John's wort, garlic oil, *Panax ginseng* and *Ginkgo biloba*. *Drugs & Aging*.

[21] Fujita T., Kawase A., Niwa T. (2008). Comparative evaluation of 12 immature citrus fruit extracts for the inhibition of cytochrome P450 isoform activities. *Biological & Pharmaceutical Bulletin*.

[22] Bigler J., Whitton J., Lampe J. W., Fosdick L., Bostick R. M., Potter J. D. (2001). CYP2C9 and UGT1A6 genotypes modulate the protective effect of aspirin on colon adenoma risk. *Cancer Research*.

